# Online Behavioral Addictions in 2023: An Overview and Current Considerations

**DOI:** 10.1192/j.eurpsy.2023.140

**Published:** 2023-07-19

**Authors:** M. N. Potenza

**Affiliations:** Yale School of Medicine, New Haven, United States

## Abstract

**Abstract: Introduction:**

Behavioral addictions (with related behaviors often conducted online) constitute new categories within the Diagnostic and Statistical Manual (DSM) and International Classification of Diseases (ICD). In both, gambling disorder is listed as a formal clinical diagnosis whereas disorders related to videogaming exist in the DSM (research criteria) and ICD (clinical criteria). However, a broader range of disorders related to other behaviors (e.g., shopping/buying, social media use, pornography use) have been proposed as possible behavioral addictions.

**Objectives:**

This presentation will provide insight in efforts by major organizations (e.g., the World Health Organization) to consider behavioral addictions and how best to screen for, assess and intervene to help people with these conditions. Empirical data influencing the classification of the behaviors and disorders will be presented. Changes in online behaviors during the COVID-19 pandemic will be considered with respect to mental health concerns.

**Methods:**

Multiple methods ranging from results of neuroimaging studies, clinical trials, and longitudinal investigations will be presented.

**Results:**

Brain imaging results suggest similarities between specific internet-use behaviors (especially internet gaming disorder) and substance use disorders, supporting a classification as behavioral addictions. Multiple types of internet use increased during the pandemic, particularly during the onset. Different patterns of mental concerns in relation to internet use behaviors were seen during the pandemic. Consensus guidances regarding how best to avoid and address problematic use of the internet were developed and disseminated. Clinical trials support the efficacy of behavioral and neuromodulatory approaches, although no treatments have regulatory approval for behavioral addictions.

**Conclusions:**

Multiple internet-use behaviors may form the basis of behavioral addictions. While considerable data exist for internet gaming disorder, other behaviors commonly performed on the internet also warrant consideration. Additional research is needed to develop, test and implement more effective prevention and treatment strategies.

**Disclosure of Interest:**

None Declared

